# The Insulin-Sensitive Side of SHIP2

**DOI:** 10.1100/tsw.2001.34

**Published:** 2001-05-11

**Authors:** Stephane Schurmans

**Affiliations:** IRIBHN (Institute of Interdisciplinary Research) at IBMM (Institute of Molecular Biology and Medicine, Gosselies, Belgium

**Keywords:** SHIP2, lipid phosphatase, 5-phosphatase, type II diabtes mellitus, knock-out mice, insulin

## Abstract

A substantial and increasing proportion of death and disability in the EU (and elsewhere) is attributable to diseases associated with insulin resistance (i.e., decreased insulin sensitivity). Beside type II diabetes, other diseases like obesity, hypertension, atherosclerosis, hyperlipidaemia, polycystic ovarian syndrome, and acromegaly are indeed associated with insulin resistance [1].

Type II diabetes is considered as the most common metabolic disease worldwide. Every day, for example, 1700 new cases of diabetes are diagnosed in the U.S., and more than 100 million people are affected worldwide. It is estimated that by the year 2020, there will be ~250 million people affected by the disease [2]. Diabetes is a major cause of blindness, renal failure, and lower limb amputations in adults, and patients suffering from the disease are at high risk to develop cardiovascular and neurovascular complications (myocardial infarction, stroke).

Type II diabetes is characterized by peripheral insulin resistance (in muscles, adipose tissue, and liver) *and* defect in insulin secretion by pancreatic beta cells [3,4]. Environmental (diet, sedentarity, age) as well as genetic factors interact to affect disease prevalence.

On the contrary, type I diabetes mellitus results from a specific autoimmune attack and destruction of the insulin-producing pancreatic beta cells; it accounts for less than 10% of the total diabetes cases. Binding of insulin to its receptor at the surface of muscle, liver, or adipose cells initiates a series of events leading to glucose uptake by the cell. Failure to properly transmit the insulin signalling (i.e., insulin resistance) results in a decreased glucose uptake. An increased blood glucose concentration (hyperglycaemia) may follow if the secretory capacity of pancreatic insulin-producing cells are no more able to compensate for the insulin resistance. Chronic hyperglycaemia then leads to the vascular and neuronal complications of the disease.

A recent study published in *Nature* has identified an enzyme called SHIP2 (for SH2 domain-containing inositol 5-phosphatase type II) as a potent negative regulator of insulin signalling and sensitivity *in vivo* [5]. Indeed, complete loss of SHIP2 gene in knock-out mice leads to increased insulin sensitivity, which is characterized by severe neonatal hypoglycaemia, deregulated expression of genes implicated in gluconeogenesis, and perinatal death. Moreover, adult mice that are heterozygous for the SHIP2 mutation and thus express only *reduced* amount of the enzyme also have increased glucose tolerance and insulin sensitivity, although still viable. Together, these *in vivo* results suggest that the enzyme SHIP2 acts as a potent brake on the intracellular insulin signalling.

Studies on the early cellular events following binding of insulin to its receptor have revealed that at least two main cascades are activated: the ras/MAP kinase and the phosphoinositide 3-kinase. The former seems more implicated in the proliferative response of the cell to insulin, and the later in the metabolic response. The phosphoinositide 3-kinase generates phosphatidylinositol 3,4,5-triphosphate (PIP3), a membrane lipid that plays a key role in this cascade by recruiting and activating proteins like PDK1, Akt/PKB, and atypical PKC, all proteins responsible for the downstream transmission of the insulin signal [6].

SHIP2 is a member of the inositol polyphosphate 5-phosphatase family; it removes a phosphate from the 5′ position of PIP3, leading to a product called PIP2 (phosphatidylinositol 3,4-bisphosphate) [7,8,9]. From *in vitro* and *in vivo* results, it seems that SHIP2 keep the production of PIP3 under control, preventing too high amount of this lipid to be produced in response to insulin. Indeed, removing SHIP2 gene as in the knock-out mice increases insulin sensitivity most probably by preventing PIP3 to be transformed in PIP2, which, in this system, does not seem to play a very important role [5]. Alternatively, overexpression of SHIP2 in cultured cells leads to decreased insulin sensitivity [10].

Together, these *in vivo* and *in vitro* results on SHIP2 imply that this enzyme represents a nice therapeutic target for the treatment of insulin resistance, and particularly type II diabetes. Indeed, molecules that decrease the activity and/or the expression of SHIP2 should increase insulin sensitivity, and restore a normal sensitivity in insulin resistant people. The availability of high throughput screening (HTS) tests to identify such molecules that interact with SHIP2 is the next step in the development of new antidiabetic drugs based on the SHIP2 results.

A second implication of these results is that SHIP2 represents a new candidate gene for the predisposition to type II diabetes. From studies in type II diabetic patients and rat models, a clear role for genetic factors have been demonstrated in the pathogenesis of the disease, beside environmental factors like obesity. Indeed, alteration in the SHIP2 gene that results in an increased activity and/or expression of the enzyme should result in an decreased insulin sensitivity, a hallmark of the disease. Sequencing of the SHIP2 gene in diabetic patients and controls should rapidly give the answer to the question.

| | | |
|---|---|---|
| Received: | February | 8, 2001 |
| Accepted: | March | 26, 2001 |
| Published: | May | 11, 2001 |
